# First Report of Entrectinib as a Treatment Option for Pure Squamous Cell Carcinoma Harboring *ROS1* Rearrangement: Exploring the Role of Next-Generation Sequencing in Targeted Therapy

**DOI:** 10.3390/ijms27010025

**Published:** 2025-12-19

**Authors:** Yan-Jei Tang, Rung-Hsuan Chen, Yung-Shin Lu, Chiao-En Wu

**Affiliations:** 1 College of Medicine, Chang Gung University, Taoyuan 333, Taiwan; c4059802@newcastle.ac.uk (Y.-J.T.); sharon.881216@gmail.com (R.-H.C.); luyungshinno@gmail.com (Y.-S.L.); 2 Division of Hematology-Oncology, Department of Internal Medicine, Chang Gung Memorial Hospital at Linkou, Taoyuan 333, Taiwan; 3 Department of Postgraduate Year Intership, Chang Gung Memorial Hospital at Linkou, Taoyuan 333, Taiwan; 4 Division of Hematology-Oncology, Department of Internal Medicine, New Taipei Municipal TuCheng Hospital, New Taipei City 236, Taiwan

**Keywords:** *ROS1* rearrangement, entrectinib, squamous cell lung cancer, non-smoking, next-generation sequencing, non-small cell lung cancer

## Abstract

Lung cancer remains the leading cause of cancer-related mortality worldwide, with non-small cell lung cancer (NSCLC) accounting for the majority of cases. Among the NSCLC subtypes, squamous cell carcinoma (SCC) is less frequently associated with actionable genetic alterations. Herein, we present the first known case of *ROS1* rearrangement in pure SCC, identified using next-generation sequencing (NGS), and successfully treated with entrectinib for approximately one year. This case highlights the potential of *ROS1* as a therapeutic target in SCC, which has historically been considered rare, as *ROS1*-rearranged SCC accounts for only 0.2% according to the Foundation Medicine database. This underscores the importance of incorporating NGS into clinical practice, particularly for never smokers/light smokers or patients with advanced SCC of the lungs, to identify targetable mutations and guide personalized therapy.

## 1. Introduction

Lung cancer is the most common cancer worldwide and is classified into two main types: small cell lung cancer and non-small cell lung cancer (NSCLC) [1]. In 2020, it was estimated that approximately 65% of global lung cancer cases occurred in males and 35% in females. Notably, lung adenocarcinoma accounts for 39% of lung cancer cases in males and 57% in females, while squamous cell carcinoma (SCC) accounts for 25% of cases in males and 12% in females [2]. In Taiwan, lung cancer has been the leading cause of cancer-related deaths since 2010, with a 5-year survival rate of 25% [3]. Between 1997 and 2016, adenocarcinoma emerged as the predominant histological type of lung cancer, accounting for nearly 90% of cases in females and over 50% in males. SCC was another type of cancer diagnosed in males but was relatively rare in females [4].

Next-generation sequencing (NGS), a revolutionary tool in biological science, has had a positive impact on personalized treatment strategies, early detection, treatment selection, and disease monitoring [5]. NGS has well-established advantages in terms of accuracy and speed compared to traditional methods, such as Sanger sequencing and Max-Gilbert sequencing, as well as enables comprehensive genomic profiling [6]. It has been widely used to detect druggable tumor-specific genes such as *EGFR*, *ALK*, *ROS1*, *BRAF*, *KRAS*, *NTRK1/2/3*, *ERBB2*, *RET*, and *MET* in patients with NSCLC [7,8].

Among the different oncogenic drivers of NSCLC, *ROS1* (ROS proto-oncogene 1, receptor tyrosine kinase) is recognized as a potential oncogenic driver [9]. *ROS1* rearrangement was confirmed as an oncogenic driver in patients with NSCLC, identified in 1–2% of patients, particularly those with adenocarcinoma [10]. However, *ROS1* is extremely rare in SCC, accounting for only 0.2% [11]. The U.S. Food and Drug Administration (FDA) has approved crizotinib, entrectinib, and repotrectinib as standard treatments for patients harboring *ROS1* rearrangement [12,13].

The Clinical Practice Guidelines of the European Society for Medical Oncology (ESMO) recommend mandatory testing for oncogenic drivers with approved targeted therapies, including *ROS1* rearrangements. In addition, ESMO advises that comprehensive molecular profiling—such as NGS—should be considered in patients with squamous NSCLC who are never smokers or light smokers, as this group has a higher likelihood of harboring actionable driver alterations [14]. In patients with NSCLC, NGS is primarily applied to lung adenocarcinoma but not SCC, given its lower incidence rates. In this case report, we discuss a non-smoking female patient diagnosed with pure SCC with an EZR-*ROS1* rearrangement detected using NGS, who was successfully treated with entrectinib.

## 2. Case Presentation

**Figure 1:**
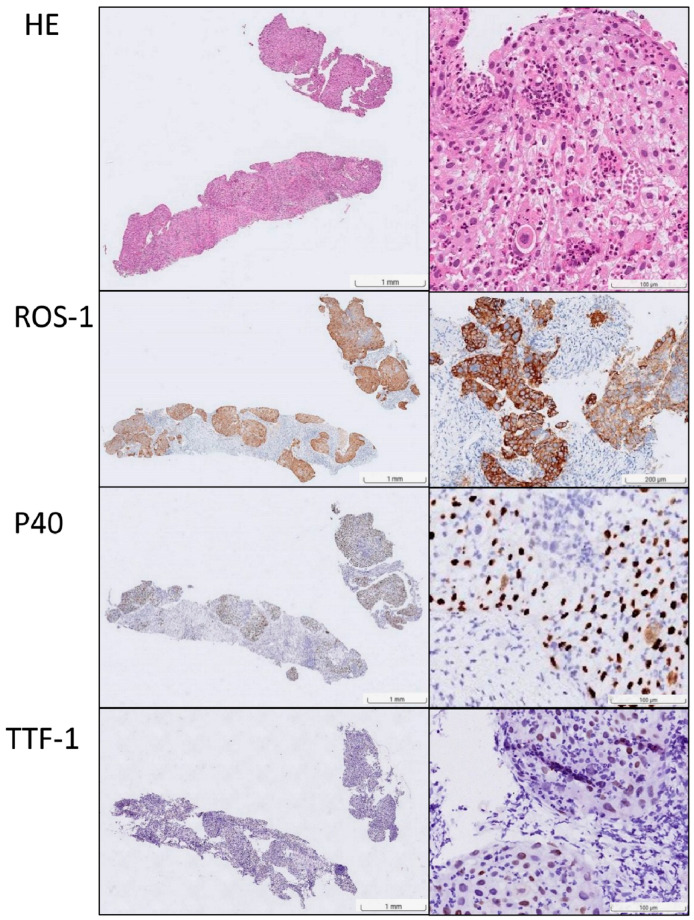
Histopathological and immunohistochemical findings supporting the diagnosis of *ROS1*-rearranged lung squamous cell carcinoma. Hematoxylin and eosin (H&E) staining shows tumor nests with characteristic squamous morphology. Immunohistochemical analysis demonstrates diffuse, strong nuclear positivity for p40 and cytoplasmic positivity for *ROS1*, with one area showing particularly strong staining. These findings confirm squamous differentiation and *ROS1* rearrangement, respectively. In addition, the absence of TTF-1 expression excludes lung adenocarcinoma. (The left column shows low-power magnification views, and the right column displays corresponding high-power magnification views.) Note: To rule out other potential etiologies, additional staining was performed. CD117 was negative, which served to exclude metastatic thymic squamous cell carcinoma. p16 staining was equivocal, a finding that argues against metastatic HPV-associated squamous cell carcinoma.

**Figure 2:**
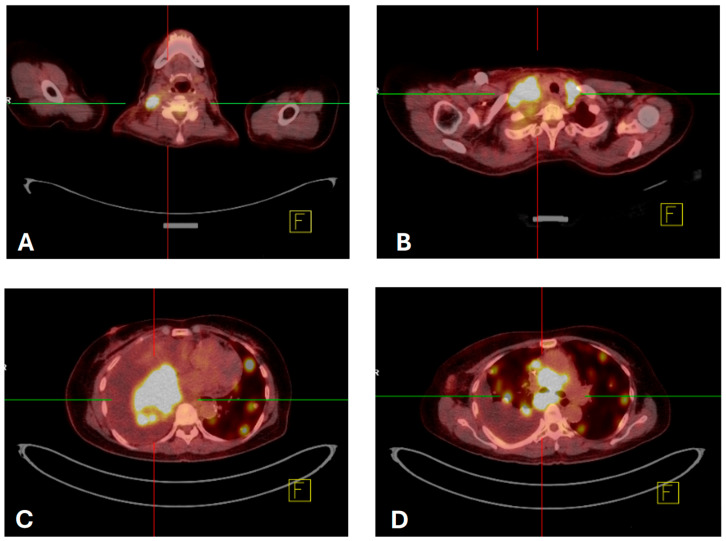
ET/CT scans of the patient showing high FDG uptake, mainly in the right lung with lung-to-lung and distant neck nodal metastasis, performed on 18 January 2024. Round high-density lesions can be observed in the following locations: (**A**) enlarged lymph nodes in the right neck, (**B**) enlarged lymph nodes in the bilateral supraclavicular fossa and mediastinum, (**C**) primary right lung tumor, and (**D**) diffuse round high-density lesions in both lungs, indicating lung-to-lung metastasis.

**Figure 3:**
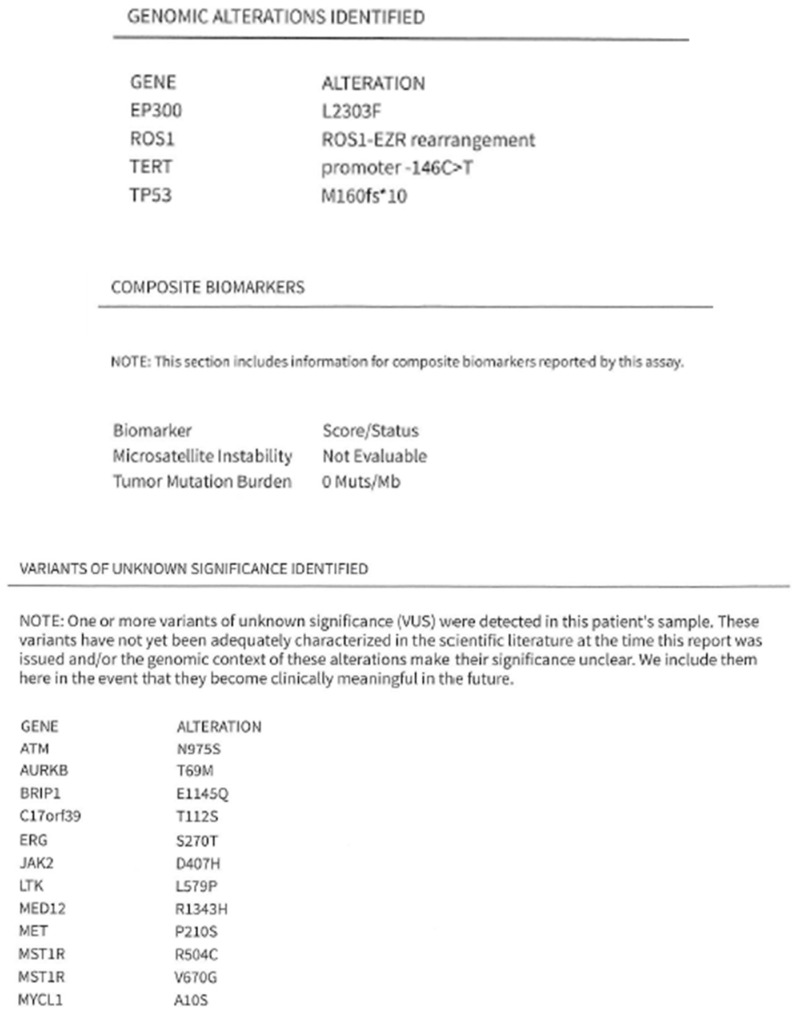
Next-generation sequencing identified several genomic alterations, including *ROS1*, EP300, TERT, and TP53, confirming that the tumor harbored a *ROS1* rearrangement. The asterisk (*) is the commonly accepted notation for a stop codon in protein mutation nomenclature.

A non-smoking 70-year-old female experienced coughing and production of white mucus for four weeks, accompanied by progressive shortness of breath. She had a history of hypertension, hyperlipidemia, and type 2 diabetes mellitus. Her Eastern Cooperative Oncology Group (ECOG) performance status was 3. Physical examination revealed a palpable mass in the right lower neck. Chest radiography revealed patchy opacity in the right medial lobe. Her symptoms worsened the following month. Chest computed tomography (CT) revealed diffuse round high-density lesions in both lungs, pleural effusion on the right side, obstructive pneumonitis, and lung-to-lung metastasis, accompanied by multiple enlarged lymph nodes in the right neck, bilateral supraclavicular fossa, and mediastinum and no brain metastasis in December 2023. Pathological examination of an ultrasound-guided core needle biopsy specimen of the right neck lymph node revealed metastatic SCC (Figure 1). Pleural effusion fluid cytology identified metastatic SCC with atypical keratotic and epithelioid cells. Immunohistochemical analysis confirmed the presence of p40/p16-positive, CD5/CD117-negative SCC. Positron emission tomography (PET)-CT revealed right lung cancer with multiple nodules, pleural effusion, lung-to-lung metastasis, and distant nodal neck metastasis (Figure 2). Accordingly, the patient was clinically diagnosed with stage IV lung squamous carcinoma (T4N3M1b according to AJCC 8th). NGS (FoundationOne Liquid Dx, Cambridge, MA, USA) identified genomic alterations, including *ROS1*, EP300, TERT, and TP53, revealing that the tumor harbored an EZR-*ROS1* rearrangement (Figure 3).

**Figure 4:**
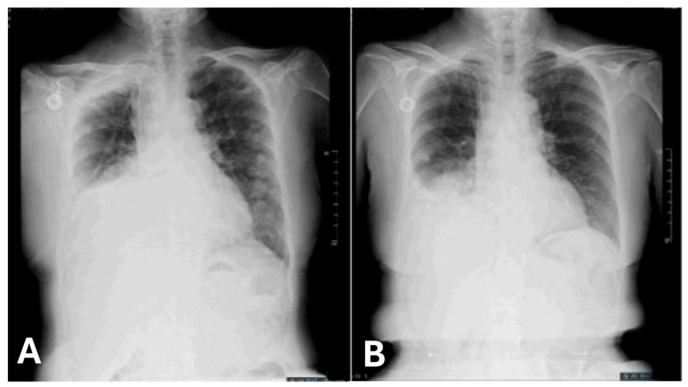
Timeline of the patient’s chest x-ray, showing a right-sided massive pleural effusion and an excellent response to continuous treatment: (**A**) 12 January 2024, and (**B**) 4 March 2024.

Chest radiography revealed progressive changes in the right pleural effusion, possibly due to lung cancer (Figure 4A). Prior to the NGS report, the patient underwent chemoimmunotherapy with pembrolizumab, paclitaxel, and cisplatin in the first cycle, based on the KEYNOTE-407 study [15]. Palliative radiotherapy to the right supraclavicular fossa, mediastinally enlarged lymph nodes, and right hilar mass comprised 40–50 Gy in 15–20 fractions, initiated at approximately the same time. One week later, the NGS results revealed that the tumor harbored an EZR-*ROS1* rearrangement, which was further confirmed by positive *ROS1* immunohistochemical staining. Treatment with entrectinib 600 mg daily was initiated on 2 February 2024. Chest radiography demonstrated improvement in the pleural effusion (Figure 4B). Following continuous treatment, the initial Eastern Cooperative Oncology Group performance status (ECOG PS) of 3 improved to 2–3 a month later.

**Figure 5:**
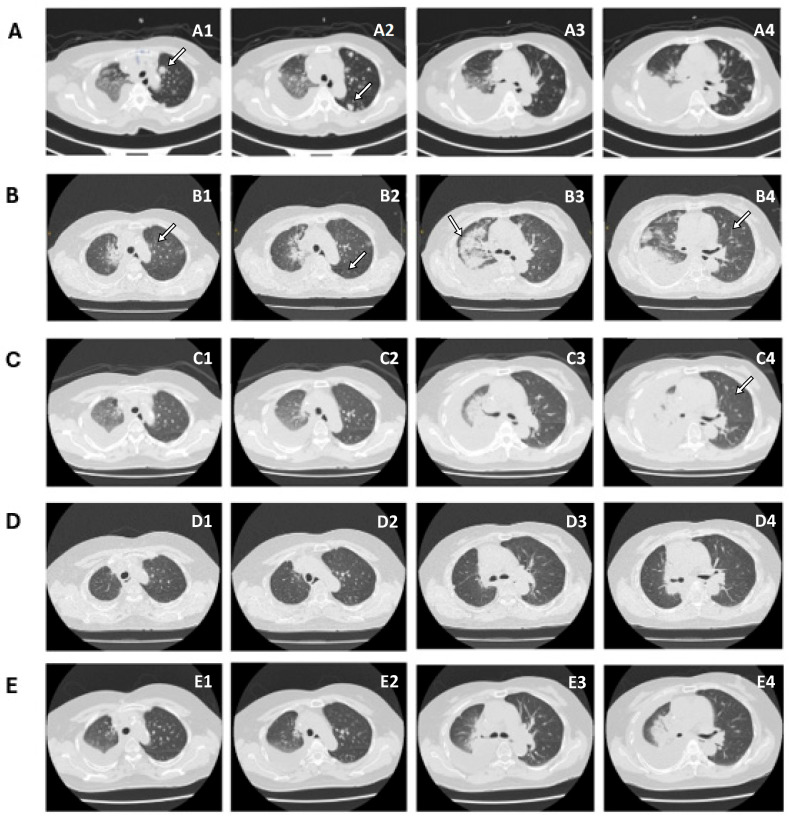
Serial follow-up CT imaging after entrectinib administration demonstrates improved outcomes. (**A**) CT imaging shows the initial diagnosis performed on 18 January 2024. (**B**) Compared with the prior PET-CT, a marked regression of the primary right lung tumor and left lung metastases can be observed, particularly evident when comparing A1 with B1 and A2 with B2. Residual mediastinal and right hilar lymphadenopathy (station 4R: 2.5 → 1.7 cm; station 7: >2 → 1.1 cm), as well as left lung metastases (largest in Left lower lung(LLL): 1.6 → 0.9 cm), appear smaller and fewer. Post-radiation pneumonitis and treatment-related changes are visible on the 22 April 2024 image B3. A further reduction in mediastinal lymph node burden is clearly appreciated when comparing images B4 and C4. (**C**) Bilateral pulmonary metastases (<1 cm) and metastatic mediastinal lymph nodes (largest: 1 × 1 cm in the left paratracheal area) are slightly smaller and fewer in number on 25 September 2024. (**D**) Metastatic lymph nodes in the bilateral mediastinum and bilateral lung metastases appear reduced in size and number on 12 December 2024. (**E**) Right lung cancer status post concurrent chemotherapy(CCRT), with partial regression, on 17 March 2025.

Compared with CT scans performed on 18 January 2024 (Figure 5A), CT scans performed on 22 April 2024, revealed notable regression of the primary right lung cancer and lung-to-lung metastases in the left lung. Residual lymphadenopathy and metastases were substantially reduced (Figure 5B). No gross bone metastases or metastatic tumors in the brain were detected. The patient is currently under follow-up and continues treatment with entrectinib, with no specific adverse events documented, including weight changes, dysgeusia, or fatigue.

## 3. Discussion

In this case report, we present a 70-year-old non-smoking female diagnosed with pure squamous NSCLC harboring an EZR-*ROS1* rearrangement who was sensitive to entrectinib. To the best of our knowledge, this is the first reported case of an EZR-*ROS1* fusion in pure squamous cell lung carcinoma successfully treated with entrectinib. Accordingly, *ROS1* can be considered a sensitive target for entrectinib in lung SCC. Typically, *ROS1* rearrangements are extremely rare in lung SCC, accounting for only 0.2% of cases. Nevertheless, oncologists should recognize *ROS1* rearrangements in lung SCC because targeted therapy may be an effective option for these patients. NGS confirmed that the tumor harbored a rare oncogenic EZR-*ROS1* fusion, and immunohistochemical examination further supported the diagnosis of SCC with this uncommon genetic alteration.

Considering *ROS1* fusion partners, EZR-*ROS1* rearrangements have been identified as the second most common. In a retrospective study of 6235 patients with advanced NSCLC (stage IIIB–IV) from five hospitals in China, 106 patients with *ROS1* rearrangements were identified using NGS. Among these 106 patients, 103 had adenocarcinoma, while only three had SCC. The authors found that the most common fusion partners were CD74 (49.1%), EZR (17%), SDC4 (14.2%), and TPM3 (4.7%) [16].

To date, only eight cases of lung SCC with *ROS1* rearrangements treated with crizotinib have been reported, all of which showed remarkable clinical improvement (Table 1). Among the eight reported cases of *ROS1* fusion-positive SCC of the lungs, five occurred in female never smokers aged between 41 and 84 years [11,17,18,19,20]. Three of the five females were of Asian origin, whereas the origins of the other two were unknown. Two cases involved a 51-year-old former-smoking female and a 71-year-old current-smoking male [21]. The remaining patients were of unknown age, sex, and smoking status [22]. Accordingly, it can be inferred that lung SCC with *ROS1* mostly occurs in non-smoking females across a wide age range. Although cases of *ROS1* rearrangements in patients with SCC are extremely rare, these cases suggest that crizotinib may be an effective treatment option. These findings highlight the importance of molecular testing for squamous NSCLC, especially in nonsmokers, to identify patients who may benefit from targeted therapy.

NGS is primarily applied in advanced non-squamous NSCLC, where tumor NGS testing is routinely recommended owing to the presence of numerous actionable genetic alterations, including EGFR, ALK, and *ROS1*, which can be targeted with specific therapies. *ROS1* fusion, classified as an IB category alteration according to the ESMO Scale of Clinical Actionability for Molecular Targets score, has strong clinical evidence supporting its therapeutic relevance [23]. The ESMO recommends tumor NGS for patients with advanced cancer in regions where tumor-targeted therapies are available. Although SCC is not typically the primary focus of NGS testing, it may be considered when targeted treatments are available.

SCC predominantly occurs in smokers and is characterized by a high frequency of mutations [24]. The application of NGS has identified common genetic alterations in squamous cell lung cancer, including TP53 (64.5%), PIK3CA (28.5%), CDKN2A (24.4%), SOX2 (17.7%), and CCND1 (15.8%) [25]. Although the most frequent mutation, TP53, remains unactionable, ERBB mutation, FGFR1 amplification, and PI3K abnormalities detected using NGS are potentially actionable genetic alterations in SCC [26].

In 2019, another SCC genomic study revealed the comprehensive landscape of genomic alterations in patients with lung SCC, highlighting both common and potentially targetable mutations [27]. Frequently observed mutations in lung SCC include TP53 (79%), CDKN2A deletions or mutations (25%), and alterations in the PI3K pathway, ARID1A, and NF1 genes. Of 130 lung SCC patients with evaluable NGS results, 38% of patients had at least one alteration that qualified them for enrollment in the LungMAP treatment arm, an approved therapy, or another clinical trial [28]. Notably, some lung SCC samples exhibited mutations typically associated with lung adenocarcinoma or other non-squamous histology, such as KRAS, EGFR, and MET mutations. These findings underscore the complexity of the histological classification of lung cancer and suggest the presence of adenosquamous carcinoma components [29]. NGS plays a key role in evaluating the molecular profile and determining the optimal treatment choice for patients with lung SCC.

The prevalence of targetable alterations also varies according to smoking history, with higher enrichment in never smokers or light smokers than in moderate or heavy smokers [30]. Upon classifying smokers as “moderate/heavy smokers (>10 pack-years)” and “light/never smokers (<10 pack-years)” [27], 47% of patients with lung SCC were never smokers or light smokers, with at least one targetable alteration that would make them eligible for FDA-approved therapy, clinical trials or off-label therapy, or LungMAP protocol therapy. After excluding six patients whose diagnostic results suggested metastatic cutaneous SCC or mesothelioma, the frequency of targetable oncogenic driver genes in nonsmokers was higher, reaching 73%. Although targetable alterations were also detected in smokers, the proportion was slightly lower than that in nonsmokers (35% vs. 47%).

In summary, this study suggests that light or never smokers with lung SCC may harbor potentially targetable genomic alterations. This finding strongly supports the current guidelines that recommend molecular profiling for squamous NSCLC in individuals classified as light or never smokers [31].

## 4. Conclusions

In conclusion, we present the first case of a non-smoking patient with pure lung SCC harboring a *ROS1* rearrangement who demonstrated a positive response to entrectinib. This case highlights the successful treatment of *ROS1* rearrangement in lung SCC and the importance of NGS in clinical practice.

Although current molecular testing primarily focuses on non-squamous NSCLC, further research on the molecular characteristics of lung SCC and the potential value of NGS in this population may be crucial for improving the treatment and prognosis of patients with SCC. NGS should be performed as early as possible in light/never smokers with SCC or patients with advanced lung SCC to improve outcomes and survival rates.

## Figures and Tables

**Table 1:** Characteristics of *ROS1* fusion gene-positive patients. (F: female, M: male, PR: partial response, CR: complete response).

Authors	Gibelin et al. [17]	Li et al. [18]	Ju et al. [19]	Yakobson et al. [11]	Yang et al. [20]	Davies et al. [21]		Shaw et al. [22]	Present Case
Sex/Age	F/41	F/45	F/84	F/63	F/47	F/51	M/71	Unknown	F/70
Race	Asian.	Asian.	Asian.	Unknown.	Unknown.	Unknown.		Unknown	Asian
Stage	Unknown	IV	III	Unknown	IV	III	I	Unknown	IV
Smoking status	Never	Never	Never	Never	Never	Former	Current	Unknown	Never
*ROS1* fusion	EZR-*ROS1*	GPRC6A-*ROS1*	Unknown	EZR-*ROS1*	Unknown	SLC34A2-*ROS1*	CD74-*ROS1*	Unknown	EZR-*ROS1*
Responses	PR	PR	PR	CR	PR	Unknown		Unknown	PR
PFS (months)	10+	5	Unknown	42+	9+	Unknown		Unknown	16+
OS	Unknown	Unknown	Unknown	Unknown	Unknown	Unknown		Unknown	Unknown
Treatment	Crizotinib								Entrectinib

## Data Availability

Data presented in this study are available on request from the corresponding author.
